# Multidisciplinary perspectives on personalised prevention in youth mental health

**DOI:** 10.3389/fdgth.2025.1568472

**Published:** 2025-10-15

**Authors:** Johanna Löchner, Mariana Bolivar, Lesley Booth, Sara Canella, Michele Calobro, Joseph Firth, Azucena Garcia-Palacios, Aleksandra Kyritsaka, Lasse B. Sander, Caroline Seiferth, Lennart Seizer, Maree Teesson, Joanna Tyrowicz, Lea Vogel, Emily Wheeler, Jörg Wolstein, Björn W. Schuller

**Affiliations:** ^1^ Department Clinical Psychology and Psychotherapy for Children and Adolescents, Friedrich-Alexander University Erlangen-Nürnberg, Erlangen-Nürnberg, Germany; ^2^ Deutsches Zentrum für Psychische Gesundheit, Berlin-Potsdam, Germany; ^3^ MQ Mental Health Research, London, United Kingdom; ^4^ NSBproject, Venice, Italy; ^5^ European Regional and Local Health Authorities, Brussels, Belgium; ^6^ Division of Psychology and Mental Health, University of Manchester, Manchester, United Kingdom; ^7^ Basic Psychology, Clinical Psychology and Psychobiology Department, Universitat Jaume I de Castellón, Castelló de la Plana, Spain; ^8^ Institute of Entrepreneurship Development, Larisa, Greece; ^9^ Medical Psychology and Medical Sociology, Faculty of Medicine, University of Freiburg, Freiburg, Germany; ^10^ Clinical Psychology and Psychotherapy, Freie Universität Berlin, Berlin, Germany; ^11^ The Matilda Centre, Sydney of University, Sydney, NSW, Australia; ^12^ University of Warsaw, Warsaw, Poland; ^13^ FAME|GRAPE, Warsaw, Poland; ^14^ Department of Law & Augsburg, University of Augsburg, Munich, Germany; ^15^ Department of Psychology, LMU-Munich, Munich, Germany; ^16^ German Center for Mental Health (DZPG), Munich, Germany; ^17^ Department of Psychopathology, University of Bamberg, Bamberg, Germany; ^18^ Chair of Health Informatics, School of Medicine and Health, Technical University of Munich, Munich, Germany; ^19^ Munich Data Science Institute (MDSI), Munich, Germany; ^20^ Munich Center for Machine Learning (MCML), Munich, Germany; ^21^ Group on Language Music and Audio, Imperial College London, London, United Kingdom

**Keywords:** prevention, mental health, artificial intelligence, adolescence, implementation

## Abstract

The pervasive impact of mental illnesses extends beyond individual suffering, affecting families, communities, and societies at large. Prevention efforts are imperative to mitigate this burden, promoting well-being and resilience across diverse populations. A particularly vulnerable period is adolescence, which is associated with numerous mental health issues that are exacerbated by declining healthy behaviours as well as socioeconomic inequalities. But adolescence also presents an opportune moment for early intervention. However, recognising warning signs and providing timely support involves considerable hurdles, so innovative prevention measures are needed. Advancements in AI, particularly in emotion recognition, offer promise for early mental health intervention. Yet, current AI achievements fall short in addressing the mental healthcare gap. This vision paper seeks to outline future directions and recommendations for effective preventive approaches by integrating experts of the necessary multidisciplinary field to develop, evaluate and implement novel and promising prevention approaches. Therefore, representatives based in Europe from diverse fields such as clinical psychology, computer science, physical activity, nutrition, economics, entrepreneurship, politics, and digital innovation propose potential avenues to integrate efficient treatment, AI methodology, and comprehensive implementation strategies that align with user needs. Based on a literature review and expert consensus, key ingredients suggested for effective preventive measures for mental health include holistic, individualised, AI-based mHealth interventions, leveraging smart and passive data from digital biomarkers for monitoring and feedback, evaluating cost-effectiveness, conducting participatory research to ensure user acceptance, and identifying barriers and facilitators for integration into regular healthcare systems. By utilising AI-driven interventions for adolescents, we can address the urgent need for preventive mental healthcare, ultimately enhancing the well-being of future generations.

## 1 Introduction

Growing up comes with several developmental challenges, with adolescence being a particularly vulnerable phase during which the incidence of mental illness peaks at the age of 14.5 years (1) and behaviours involving healthy nutrition and physical activity diminish (2). Particularly, psycho-socially disadvantaged youth with e.g., low financial resources, disruptive families, from rural areas are 3–6 times more likely to develop adverse health outcomes as obesity and mental health conditions, including addictive behaviours (e.g., excessive use of computer games) (3, 4). At the same time, adolescence also represents an excellent opportunity to prevent the onset and the potential chronification of mental and physical illness (5).

However, recognising early warning signs and offering timely support remains a major challenge. Help-seeking is hindered by numerous structural limitations (e.g., limited treatment capacities, long waiting lists) and individual barriers (e.g., stigma) (6). Hence, new measures for the prevention of mental illness are needed (7).

As a reaction, there is a growing body of research, innovation and investment from multiple sectors towards the use of digital technologies to create a physically fitter and healthier society (8). This surge in digital health interventions reflects an unprecedented integration of technology in everyday health management. Examples include the public health initiatives of text messaging campaigns to promote health behaviours (9), the increased use of “app libraries” within healthcare services and steps towards recommending health promotion apps through primary care (10), along with the rapidly-expanding commercial industry of digital health through wearable activity trackers (11). Furthermore, there is a plethora of mHealth interventions focusing on well-being and general health.

The implications of these technologies for public health are substantial. By leveraging the ubiquity of mobile phones and the possibility of granular data collection of wearables, health interventions can be personalised, timely, and contextually relevant. Such an individualised approach would have the potential to significantly improve outcomes. Thereby, the field of Artificial Intelligence (AI)—especially the assessment of individual symptoms based on automatic emotion recognition—has witnessed remarkable advancements in recent years, yet its potential remains largely untapped in the realm of recognition and early intervention for (mental) health and the application into clinical practice (12, 13). Harnessing these cutting-edge technologies presents a unique opportunity to provide individualised support to promote behaviour management and to improve quality of life (14, 15).

Unfortunately, however, it remains insufficiently examined to which degree these digital innovations for behavioural health are accessible and effective for individuals with mental health challenges, particular among youth (16–19). Addressing this gap is crucial, as early intervention can significantly alter the trajectory of young lives affected by mental health issues.

In addition, a significant gap remains between what is technologically possible in research settings and what is implemented in real-world practice—particularly for adolescents. Moreover, much of the existing evidence stems from studies with adult populations. Therefore, it is imperative for researchers, clinicians, and policymakers to direct their efforts towards translating recent advances in digital behavioural health into holistic mental health care. A holistic approach opens new pathways for addressing adverse health behaviours, improving physical health outcomes, and facilitating the prevention and treatment of mental illness, especially in young populations. Furthermore, it is crucial to design evidence-based applications and to develop sustainable implementation strategies for policy and clinical practice recognising the target group’s needs.

In this manuscript, we will make a case for holistic, personalised, AI-based preventive mHealth interventions including ongoing monitoring and feedback tools. This way, interventions allow an ever-earlier precise mental health status assessment and prevention of mental health conditions, shifting from treatment-focused to person-centred care enhancing individual growth. We target the 15–25 years age group because it represents a critical period when most mental health issues first emerge, digital engagement is high, and early intervention can have the greatest long-term impact (1). In addition, individuals from low socio-economic backgrounds and rural areas, given its strong potential to improve access to healthcare for these underserved populations. In addition, the integration of AI in health care needs to be critically evaluated and its benefits assessed for effectiveness, scalability, and cost-effectiveness. This way, we aim to highlight concrete avenues for bridging this gap by synthesising the current state of the art and outlining key steps needed to translate these advancements into effective, scalable interventions for youth mental health. Experts from diverse disciplines across Europe—ranging from fields such as clinical psychology, computer science, physical activity, representatives of lived experience experts, nutrition, economics, entrepreneurship, politics, and digital innovation joined forces through a coordinated effort to address the urgent need for AI-based prevention in mental health. This collaborative initiative was intentionally designed to integrate scientific, clinical, and technological perspectives into a unified vision for future prevention strategies. Such experts were chosen and invited to this article due to their track record in the respective field. Following a series of structured meetings, collaborative writing sessions in sub-groups of authors, and iterative feedback rounds, the group developed this paper to aggregate insights and propose a shared vision for future prevention strategies. MT, as major expert in the field of youth prevention, reviewed the final article. This paper focuses on Europe as a representative region to explore the practical implementation of AI-based mental health prevention. Europe offers a diverse yet integrated landscape of healthcare systems, legal frameworks, and cross-country collaborations, making it a valuable context for examining region-specific opportunities and challenges. While our analysis is rooted in the European setting, the underlying principles, challenges, and proposed strategies are broadly applicable and can serve as a basis for adaptation in other regions worldwide. This visionary article further aims to raise attention for research supporting young people to adopt and maintain a healthy lifestyle and improve physical and mental well-being for personal growth following a multidisciplinary approach.

Building on our group discussions, we define the following objectives to outline promising AI-based preventive approaches for youth and to highlight key considerations for their effective implementation:

I. Development of a holistic, individualised, AI-based mHealth preventive intervention for mental disorders and health behaviour featuring early and precise mental health status assessment for adolescents with psycho-social disparities; authors: LSa, CS, JF, JW, LV, JL
II. Integration of smart active and passive assessment of digital biomarkers (including physical as well as psychological parameters), ensuring continuous monitoring and personalised content covering general and mental health aspects, including a critical assessment of AI integration; authors: BS, LSe
III. Evaluation of the (cost-)effectiveness of AI-based mHealth preventive interventions through multi-site, prospective randomised controlled trials across multiple countries; authors: JT, JL
IV. Inclusion of the perspective of the users by user-needs assessments and participatory approaches for co-creation of digital interventions; authors: MB, LB, EW
V. Identification of barriers and facilitators for implementation, and devise strategies for dissemination, exploitation, and sustainable health support across Europe. authors: SC, MC, AK

Each paragraph follows a structure that includes an overview of the current state of the art, identification of research and application gaps, corresponding recommendations, and concluding remarks. However, given the interdisciplinary nature of the field and the diversity of expert perspectives, certain areas may receive greater emphasis than others.

## 2 Toward transformative prevention: a roadmap for next-generation AI in youth mental healthcare

In the following, considering the state-of-the-art in research, approaches for the development, evaluation and implementation of a successful holistic and AI-based preventive intervention are outlined.

### 2.1 A holistic, individualised, mHealth preventive intervention for mental disorders

To date, the emphasis of e-mental health has predominantly been on managing or monitoring just the psychiatric symptoms that define these conditions. However, it is important to remember that people with severe mental illness die approximately 15 years younger than the general population, mainly due to their increased risk of developing cardiovascular and metabolic diseases (20). Therefore, further research and investment should be directed towards establishing how people with mental health problems can equally benefit from digital innovations for physical health. Failing this, people with mental illness may be “left behind” from the increased use of digital technologies in physical health contexts, which then would serve to actually increase the extent of the disparities already observed.

Preliminary evidence indicates that digital health approaches can be effective in supporting holistic and behavioural health among people with severe mental health problems (21). As physical health and behaviour risk factors typically begins to deteriorate during the early stages of mental illness (22, 23), mHealth could ideally be utilised to support timely preventative health interventions in young people at risk of mental illness (22), to reduce or delay the onset or exacerbation of comorbid physical health conditions (24, 25). Beyond physical benefits, digital interventions targeting holistic factors such as exercise (26) and sleep (27) have also been shown to effectively reduce depression. Furthermore, proactive engagement with individuals experiencing chronic physical conditions has also shown to be a valuable approach in the prevention of depression (28, 29). Applying such approaches in younger populations may confer even greater benefits, given the higher levels of adoption and usage of smartphone technologies in these groups (30, 31).

Scaling up the research here warrants attention in two areas. First, discovering how physical health behaviour (such as physical activity, healthy eating, smoking cessation and better sleep) can be effectively promoted via digital technologies for people living with, or at risk of, mental illness may lead to novel interventions for improving both physical and mental health outcomes (20, 21).

Second, establishing whether and how these interventions can be implemented in ways that meet the unique needs of youth with mental illness is essential to ensure real-world adherence and effectiveness in youth mental healthcare, beyond trial conditions (30, 32). Meeting the needs of underserved populations can include adapting existing technologies, providing additional in-person support, and/or developing bespoke solutions for specific patient groups (31).

Recommendations: A holistic approach in healthcare underscores the promotion of general health and well-being, instead of focusing exclusively on preventing specific diseases or conditions. This comprehensive strategy encompasses lifestyle, stress management, nutrition, physical activity, and other elements contributing to general well-being (33). Emphasising the psychological perspective, it is essential to consider the underlying mechanisms of change involved in the development of mental illness. Conditions such as maladaptive cognitive style, emotion dysregulation, and dysfunctional interpersonal behaviour represent promising intervention targets for digital health interventions in youth (34–38). Targeting these mechanisms offers a potential avenue for more effective and efficient preventive strategies. In this context, behavior change techniques (BCTs) are active components of interventions that target underlying mechanisms and promote favourable health behaviour change (39, 40). Hence, mHealth interventions aiming to improve participant health outcomes (i.e., weight, mental well-being, nutrition behaviours, physical activity) across the lifespan increasingly incorporate BCTs such as self-monitoring, goal setting, social support and feedback on behaviour and performance (41), and seem to be promising in preventive digital interventions in children and adolescents (42). However, it is noteworthy that a large body of studies evaluating the efficacy of behavioural interventions and using BCTs has focused primarily on adolescents with a high socioeconomic status (SES), and a high level of education (42). Future research should explore the effectiveness of BCTs among adolescents with ethnic minority backgrounds and low SES to ensure effectiveness in diverse populations. Here, evaluating usability, usefulness, and usage together with people with lived experience can inform the creation of more effective, high-quality, and user-friendly digital health approaches (43).

To overcome such health inequalities, the application of less intrusive methods that are attractive for youth, such as smart active and passive data collection with individual feedback, may support the integration of young people from less advantage backgrounds.

### 2.2 Smart active and passive assessment of digital biomarkers for mental health

To offer personalised preventive interventions, it is crucial to capture the psychological state of an individual. Hence, as a foundation, it is necessary to collect reliable data. In case such data is inaccurate or misleading, personalised interventions risk being ineffective or even counterproductive. Most phenomena that are of interest in mental health are, for once, heterogeneous across individuals, and secondly not static but rather highly dynamic (44). Still, medical research and clinical decision-making mostly rely on nomothetic (cross-sectional and group-aggregated) results that can provide valuable information on a population level but often fail to do so on the level of individual patients (45, 46). Naturally, behaviours, states, and symptoms vary across situations and time; and in clinical practice, the focus is often on understanding their dynamics and contexts, i.e., when and under what circumstances symptoms occur. For instance, depressive episodes involve persistent low positive emotions, borderline personality disorder is associated with unstable emotions, and compulsions often arise when anxiety levels increase (47). Mental health symptoms develop from a complex interplay between individual and environmental factors, and it is essential to capture these fluctuations over time to identify risk factors, trajectories of illness, and opportunities for intervention (48).

In the last decade, the rise of deep learning has led to major advances in the broader field of affective computing, which deals with the analysis, synthesis, and response to human affects and emotions (49). In this regard, data from the domains of audio (e.g., speech), video (e.g., facial expression, body posture, gait), text (written or spoken language), physiology (e.g., via heart rate, skin conductance, EEG and brain computer interfaces), and tactile and further contextual and interaction data serve as the main means of assessment. In the mental health context, AI has been used to detect emotions and mental illnesses, such as depression and bipolar disorder, through audio-visual data for example by spoken language and facial expression (50–52). Speech signals have thereby been shown to contain depression-relevant information for machine learning analysis both in terms of their acoustics and linguistic (spoken) content (53, 54). In addition, EEG data have been successfully used for emotion recognition (55) and applied, e.g., in depression recognition (56). However, since not all parts of a signal will exhibit markers of depression to equal degrees, attention mechanisms can be introduced to allow neural networks to learn salient parts of the input (57, 58). Further, transfer learning can be considered a fruitful strategy to tackle the often limited availability of training data for the target task (59, 60).

Passive measurement methods offer a convenient approach to data collection that requires minimal effort from participants which may increase the acceptance and adherence by youth. Wearable devices, discreetly worn on the body, continuously gather data through sensors, seamlessly integrating into everyday activities. In a literature review, Wac (61) identified 438 distinct wearable sensor devices. These devices are typically worn on the wrist, head, torso, chest, ear, or arm, and are capable of measuring an extensive array of bio-physiological parameters. Moreover, advances in mobile neuroimaging techniques further enhance our understanding by allowing neural responses to be measured in real-world settings and enable unprecedented insights into everyday neural dynamics (48), e.g., portable magnetic encephalogram caps (62) and mobile deep brain recording (63). Environmental factors can be captured through tools like audio recorders, such as the electronically activated recorder (EAR), that record ambient sounds as participants navigate their daily lives (64). Similarly, video or image capturing can give insight into the facial expression and facial micro expressions. Smartphones represent a particularly promising ambulatory technology, as they comprise a range of sensors that gather data on geolocation, app usage, communication patterns, and social media activity. Further information is collected such as the Near Field Communication (NFC) data that gives insight whether users are likely among other individuals or alone, or the light sensor data usually used to control display brightness, yet also giving insight on whether individuals are exposed to light or literally “remain in the dark.” This wealth of information enables the inference of behavioural states through digital phenotyping (65). Incorporating such sensor data into smartphone apps is an effective way to provide additional (objective) evaluations of a person’s mental well-being (66). For example, the authors in (67) used smartphone sensing data to detect users’ self-rated compound emotional state and achieved accuracies of over 70%. A study carried out in 83 undergraduate college students aimed to quantify depression symptoms by measuring phone use, heart rate, sleep, and location using a wrist sensor and a smartphone app (68). Depressed students were found to use their phone more often in study locations, had irregular sleep patterns, and were more stationary.

In addition to such passive data, Ecological Momentary Assessment (EMA) via smartphones offers the opportunity to report variables in patients’ everyday lives continuously and unobtrusively. Frequent assessment is vital as mental health is dynamic, influenced by emotions, cognition, and behaviour, and subject to affective states and contextual variables (44, 69, 70). This requires regular monitoring to capture changes and anticipate transitions (71–73). In this regard, EMA has significantly transformed the collection of behavioural and experiential data in natural environments and has addressed issues such as biased recall-based assessments and noncompliance with participant obligations, thereby improving the inclusiveness and comprehensiveness of research efforts (74). The introduction of smartphone-based EMAs enables the collection of ecologically valid data in real time, providing deep insights into phenomena such as mood swings and sleep patterns. This is particularly beneficial for populations such as children and adolescents, where retrospective reporting may be less reliable (75). The integration of both, passive data (wearable sensors, audio recordings) and active data (questionnaire items on mood, sleep, stress), facilitated by smartphones, represents a significant advance in understanding behaviour in real-world contexts (12). Note that EMA has also been used to actively collect other data forms such as free speech (76). This integration helps to develop personalised psychiatric treatments, as it offers more precise insight into behavioural and environmental patterns.

Despite the rapid development of innovative technologies for detecting emotions and other health-related variables, research focusing on adolescents is often constrained by small sample sizes and primarily involves individuals who are already depressed (77–81). These studies typically use sensor data collected from smartphones and wearables, including information such as movement, light, and various health metrics (81). Preliminary findings suggest that sensing data can effectively predict depressive symptoms, with correlations between sensing parameters and depression scores ranging from 0.44 to 0.72 (77, 80). The challenges encountered in these studies include battery life issues, data usage, and privacy concerns (79). In a study with 122 adolescents with and without depression, significant correlations have been observed between sensor data and symptoms of depression and anxiety, with high levels of acceptance reported among participants (78). These findings highlight the potential of sensing technologies in understanding and monitoring mental health in adolescents (82). However, many of the cited studies were conducted during the Covid-19 pandemic and hence have limitations regarding some sensors (e.g., mobility as predictor) and the generalisation of findings. The limited number of studies on smart sensing that focus on emotions in youth populations may reflect the challenge of developing emotional representations or competencies in children and adolescents that differ greatly from adults, especially in the vulnerable phase of puberty (83). Nevertheless, unobtrusive smartphone monitoring of psychopathological symptoms and personalised feedback has a huge potential to gain insights into an individual’s well-being while keeping effort from participants low.

Recommendation: To advance preventive mental healthcare in adolescents, we recommend developing integrated AI-based systems that combine passive sensing (e.g., wearable and smartphone data) with active methods such as Ecological Momentary Assessment. These systems should leverage multimodal data (e.g., speech, physiological signals, behaviour patterns) to detect early warning signs of mental health issues in real time. Particular attention must be given to adolescent-specific emotional expression and developmental differences. Research should prioritise large-scale, longitudinal studies with diverse populations to validate effectiveness, ensure ethical data handling, and enhance user acceptance. Tailored feedback and low-burden monitoring approaches are essential to maximise adherence and long-term impact.

However, technological advancements come with a cost. To obtain accessible solutions, especially for youth with lower SES, cost-effective solutions are a necessity.

### 2.3 Evaluating the (cost-)effectiveness of mHealth preventive interventions

Smartphone applications are known for their scalability. In the context of public health interventions, this implies that at a negligible or effectively zero marginal cost, individuals may receive the assistance they need. In addition, personal contact with the psychologist can be provided at a lower cost due to gains related to remoteness (reducing queues and costs associated with commute) and hourly flexibility (improving the availability of experts). But the key potential value of mHealth lies in its ability to prevent social cost rather than the low cost of provision. In fact, there are two perspectives on the sources of potential value of mHealth: private benefit and social benefit. Private benefits include improved well-being, closer connection with the labor market, higher income, and higher job satisfaction. With multi-arm randomisation in the functioning of mHealth, these outcomes can be causally measured for each participant to obtain the *effects* of mHealth (84), even without a control group per se (85).

With employment status and income objectively measured for each beneficiary, they also give rise to the social benefit calculus. Specifically, working individuals complete their education and move on to work, and thus pay social security contributions and taxes. In contrast, young people struggling with mental illness are more likely to drop out of school and be on the receiving end of social benefits rather than paying contributions (86). Thus, the immediate calculus of social benefits involves better educated labor force, paying taxes and, by the same token, lower social assistance pay outs. This calculus can be extrapolated using the effects estimated at the individual level among the users of mHealth. This immediate calculus has a mirror counterpart in the long-term calculus, which can only further improve the cost-benefit analysis of mHealth.

Applications such as mHealth can also introduce negative and positive externalities to participants. For the former, completing tasks aimed at improving mental health and reporting your own mental state in the app can be taxing for participants. For the latter, the intervention can rely on past patterns of lower mental status to reach out to users even before the user reports an issue and takes action in the app. Stone et al. (87) propose a daily reconstruction method to obtain adequate estimates of the emotional costs associated with completing treatment procedures on the app (88). Specifically, the mHealth intervention can provide data on the levels of stress, anxiety, fatigue, etc. associated with specific activities, probed through the app at random moments of the week among our participants. mHealth applications can internally validate the accuracy of the autonomy of the application from these measures.

Recommendation: mHealth interventions should be leveraged not only for their scalability and low marginal cost, but also for their broader societal value. Beyond private benefits—such as improved well-being, employability, and income—mHealth offers significant social returns by promoting educational attainment and labor market integration, thereby reducing reliance on social support systems. To capture these effects, mHealth programs should incorporate robust causal evaluation designs (e.g., multi-arm randomization) and track objective outcomes like employment and income. Policymakers and funders should consider both immediate and long-term cost-benefit analyses when investing in digital mental health solutions. Furthermore, interventions must balance user burden with benefit by monitoring emotional costs and adapting outreach based on passive indicators of distress. Ethical implementation should prioritize minimizing negative externalities while maximizing proactive, data-driven support.

Even the most innovative and cost-effective solution holds little value if it fails to engage the target group and leads to high dropout rates. Therefore, it is essential to prioritise users’ needs, preferences, and lived experiences in the development and implementation process.

### 2.4 Participatory approach

Active participation of young people with lived experience (YPLE) in mental health research allows to develop interventions that align with their needs and priorities (43). Genuine youth involvement goes beyond consultation—it includes shared decision-making, co-design, and leadership opportunities throughout the research process (89). Focus groups as well as youth advisory boards that are accompanying the process iteratively are recommended to receive qualitative feedback on the intervention’s content, design usability and attractivity. In addition, quantitative surveys increase representativeness of user tests. In terms of implementation, engaging YPLE to identify barriers and facilitators is essential for guiding policymakers toward health regulations that are most likely to positively impact young people (43). Such an approach is paramount in the development of AI-based mHealth preventive interventions (90). This includes practical advantages for researchers, including increased participant recruitment and retention (90). Participants are more likely to sign up to a study or remain in the study if consultation of YPLEs has happened. However, to achieve impactful, equitable and viable interventions, co-creation with YPLEs must not exist in a vacuum. For ensuring a comprehensive system of care, intervention design should also harness the collective expertise and perspectives of educational institutions, parents, health professionals, support services, researchers, policy makers and other related stakeholders will be equally important. Co-creation with YPLEs must also account for common barriers to achieve inclusive, diverse and meaningful involvement (see Table 1) (91).

**Table 1:** Barriers and solutions.

Existing barriers	How to overcome them	Example
Digital inequalities	Adopt both digital and non-digital strategies for dissemination and engagement	Social and print media for dissemination
Lack of relevant incentives	Set incentives in place which take into account the basic, psychological and fulfilment priorities from these populations, as seen by them	Unlock access to free mobile data or music streaming time for each week of engagement
Tokenism	Increase decision-making power and influence of Lived Experience advisors and participants	Participatory budgeting, voting power for lived experience advisors
Communication challenges	Increase translational efforts, allow bi-directional communication and avoid jargon	Community co-design workshops with plain language outputs, feedback loops
Stigma	Increase mental health awareness on the ground and empower others to challenge stigma	Street-level or social media campaigns co-designed with people with lived experience, peer ambassadors

*Digital inequalities*. Technology needs to be an enabler for YPLEs involvement in research. However, over-relying on digital channels and platforms to reach people with lived experience leaves behind populations that have limited—or do not have—access to technology. Instead research needs to seek underrepresented groups where they are, through the channels they use, and facilitated by the organisations they trust, using digital and non-digital dissemination strategies (91).

*Lack of relevant incentives*. For people from difficult to reach communities to participate in research, there needs to be relevant incentives in place, considering the basic, psychological and fulfilment needs from these populations. It is important to include monetary compensation, cover transportation or internet costs and promote career development. *Tokenism*. Participation can feel tokenistic if YPLEs are not empowered through the decision-making processes. YPLEs should be allowed to contribute to agenda/priority-setting, lead discussions and dialogues to facilitate communication and connect with the objectives that people care about. *Stigma*. It can take a lot of time and effort for some people to be in a place where they feel comfortable discussing mental health. A potential strategy to address this barrier is to work closely with organisations to promote dialogue, increase mental health awareness, and empower others to challenge stigma on the ground (92). *Communication challenges*. There are communication and translational barriers for YPLEs involvement in research. Language differences are a significant barrier, since most research activities and outputs are in English, and rarely translated into other languages, making it difficult for the non-English speaking world to participate in research, and benefit from it. Jargon is another frequent communication barrier. YPLEs have a crucial role to play, to ensure language is simple, accessible, allows shared understanding, and promotes knowledge exchange. Studies therefore need to investigate the long-term benefits of a holistic self-help AI-based mHealth intervention for the early recognition and more precise mental health status assessment, leading to vital, personalised prevention of mental disorders, enhancing health behaviour and mental well-being in a scalable, sustainable and cost-efficient way. By focusing on the needs of the target group with psychosocial and socioeconomic disparities, long-term adherence and satisfaction with the digital health intervention will be ensured. Success in the development of human-centred AI-based mHealth interventions could improve self-empowered disease management, the precise personalised mental health status assessment and more efficient guidance into the appropriate professional health care services. This co-creation involving multiple stakeholders is essential for the development of AI-based technology that is trustworthy, person-centred, evidence-based, and aligned with real-world needs from different contexts. It is also essential for overseeing the generation of ethical standards and regulations which are relevant and enforceable. This inclusive approach ensures that e user’s acceptance, scalability, sustainability, and impact of AI-based mHealth solutions, ultimately improving healthcare outcomes and empowering individuals to take control of their own well-being as well as defining strategies and recommendations that will ultimately create a more fertile ground for solutions to thrive across the globe. Through international collaborative networks, one can collectively work towards realising the full potential of AI in promoting adolescent health, fostering resilience and empowering young individuals to lead healthy and fulfilling lives.

Recommendations: To ensure AI-based mHealth interventions truly benefit young people with lived experience, inclusivity must be a core principle. Digital tools should not deepen existing inequalities; outreach must also occur through trusted community channels, especially for those with limited access to technology. Participation should be meaningful, with fair incentives—such as financial compensation, covered costs, and personal development opportunities. Tokenistic involvement must be avoided by empowering youth to shape priorities and lead conversations.

Stigma remains a major barrier, and partnerships with local organisations are key to creating safe spaces and promoting mental health awareness. Communication should be clear, jargon-free, and available in multiple languages to ensure accessibility and shared understanding.

**Figure 1:**
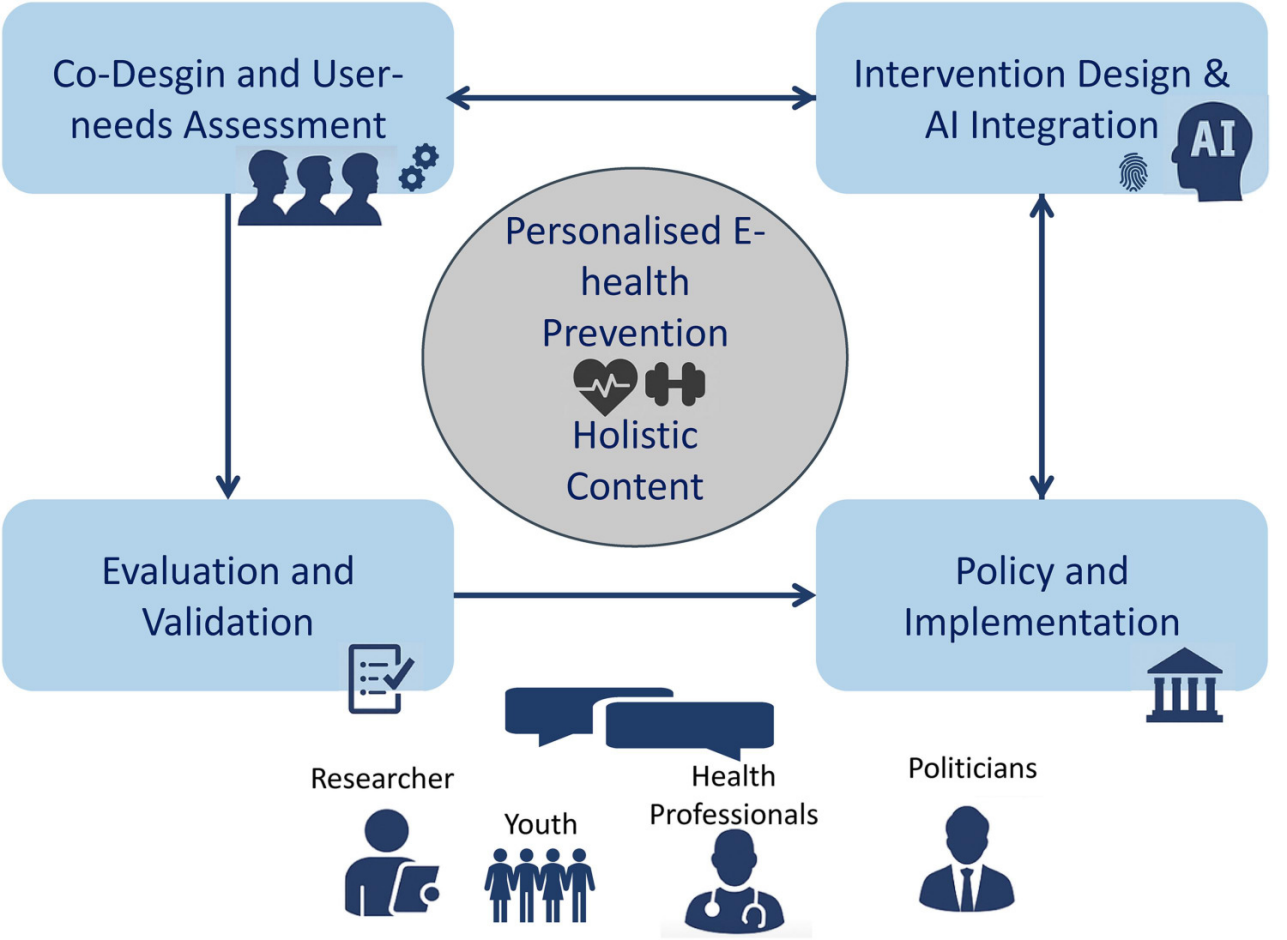
Process of iterative user-centered development, evaluation and implementation of an holistic, personalised e-health prevention for youth.

Design must be human-centred, culturally sensitive, and responsive to psychosocial and socioeconomic disparities. Co-creation with diverse stakeholders is essential to building trustworthy, effective, and ethically sound AI solutions. Finally, international collaboration is vital to scale these innovations and empower youth globally through early, personalised mental health support. Figure 1 displays the process of co-development, evaluation and implementation including youth and other stakeholders.

Finally, young people, researchers, and developers should actively engage with policymakers to identify barriers and enablers, helping to shape conditions that support the sustainable implementation and use of innovative mental health tools.

### 2.5 Barriers and facilitators for implementation, dissemination and sustainable health support across Europe

As mentioned in the introduction, there is an gap in health research between establishing the effectiveness of an intervention and its uptake in routine use (93). In the last decades, an important effort has been made to develop rigorous scientific methods and procedures to reduce such gap and increase the possibilities of citizens to have access to evidence-based practises. These methods and procedures belong to a raising discipline named Implementation Science (94), in which the field of mHealth research is an active player. The integration of AI in mHealth is poised to significantly improve mental healthcare, yet its clinical application, remains largely untapped despite the urgency for innovative solutions.

The successful implementation of e-health interventions hinges on readiness across multiple levels: individual, innovation, organisational, and system-wide (95–99). While previous research indicates that digital interventions can be effective in supporting mental health (100), contextual factors play a crucial role in determining user engagement (101, 102). To enhance individual-level readiness, ensuring equitable access to devices and providing digital literacy training is essential (97, 99). At the innovation level, making interventions more user-friendly, clinically useful, safe, and adaptable to client needs and existing clinical workflows can significantly improve their adoption (95, 97). Organisational and system readiness can be bolstered by providing adequate technology and training to providers and local behavioral health departments, and by exploring system transformations, such as integrated care models (103).

By conceptualising digital solutions as services, we can account for both, the characteristics of the innovation—efficacy and safety, as well as its clinical usefulness—and the broader ecosystem in which these interventions are deployed. This ecosystem includes individual and organisational factors (inner context), intermediaries and purveyors (bridging factors), client characteristics (outer context), and the fit between the innovation and its implementation setting (innovation factor).

The economic and societal toll of untreated mental health disorders, projected to reach 2.5 trillion USD by 2030, underscores the necessity of early and preventive interventions (104). Such interventions, especially when delivered through mHealth platforms, have proven to be cost-effective and essential for enhancing societal productivity (105).

While mHealth interventions show promise in addressing the mental health treatment gap, key barriers remain. To summarise key messages from the sections above, there is a lack of longitudinal research on their long-term impact in adolescents, particularly regarding health, education, and employment outcomes (106, 107). The global shortage of psychiatric services—especially in rural areas—underscores the need to explore how mHealth can expand access (108, 109). Additionally, AI-based mHealth tools offering personalised feedback require further validation, particularly regarding their effectiveness, acceptability, and ethical implications (110). Finally, research is limited on adolescents from non-diverse socio-economic backgrounds, leaving important questions about equity and access unanswered (111, 112).

To overcome these barriers, possible recommendations to stakeholders are focused on: (a) the development and validation of mHealth solutions, thanks to further investments in developing and rigorously testing AI-based mHealth solutions tailored for adolescents, ensuring these interventions are grounded in robust clinical evidences; (b) the increase of accessibility of services, specifically governments and healthcare providers must work together to increase the accessibility of psychiatric services, particularly in remote areas. This could involve investing in telepsychiatry and mHealth platforms as complementary solutions; (c) promotion of an early identification and intervention strategy, to prevent the escalation of mental health issues into adulthood. Schools, healthcare providers, and communities should collaborate to lower barriers to early healthcare utilisation using preventive strategies in mental healthcare, especially those leveraging mHealth technologies; (d) address socio-economic disparities through the development of policies and interventions more inclusive and considering the unique challenges faced by adolescents from varied socio-economic backgrounds. This includes tailoring interventions to be culturally sensitive and accessible to all segments of the population; increase multi-sectoral collaboration among health, education, technology, and social services, to successfully address the multifaceted nature of mental health challenges in adolescents. Furthermore, (e) the development and deployment of AI-based preventive e-health interventions for youth must align with the EU AI Act, which classifies AI in healthcare as high-risk and sets strict requirements for transparency, data quality, human oversight, and accountability. Further regulatory aspects as CE certification or European Medical Devices Regulation may set barriers due to high costs and required resources to get AI-based solutions off the leash. (f) Ethical considerations—such as safeguarding minors’ privacy, preventing bias, and ensuring explainability—are critical to building trust and protecting vulnerable populations. Compliance with these standards is essential to ensure the safe, equitable, and responsible implementation of AI-driven tools in youth mental health prevention.

Building on the analysis of the current state of the art, existing research and barriers, rapidly advancing on the application of AI in mental health for young people can be clearly considered a priority area in the overall digital and AI transformation of health and care. AI-based tools can offer a range of new opportunities addressing a series of existing obstacles, potentially providing more personalised and timely care, reaching out to a broader audience and thus delivering lower-threshold, more flexible and cost-efficient health support independent of geographical location, socio-economic and cultural background, and existing limited infrastructure or mental health provision services.

To strongly advance on this issue and ensure adoption of such innovative tools, however, it is necessary to address a series of complexities and hurdles that can be tackled only through a multifaceted approach involving for instance policy actions, education, innovation support programmes and ecosystems, ethical, security and transparency considerations. Key recommendations can be summarised as follows:

1. Increase attention on mental health overall, with a focus on adolescents—building on initiatives such as the European Comprehensive Approach on Mental Health to further increase the attention on mental health from European to local level, mobilising new resources and funding to invest in mental health education, care and innovation.
2. Investing and developing educational programmes for adolescents—aimed at both increasing health literacy and awareness on their need to freely engage in self-help health behaviours, while giving them the right information to engage with digital tools. In this framework, it will be essential to enhance collaboration with both educational and health professionals for a comprehensive approach.
3. Increased focused on co-creation with adolescents—digital tools should be developed with co-creation at their heart, to ensure that their functionalities fully respond to the needs of adolescents, thus facilitating proper adoption and engagement.

Developing policies and guidelines ensuring transparency, security and a sound ethical approach. As with all related digital health and health data-based tools, it is essential that policies and guidelines, from European to local level, will guarantee by design transparency, security and address potential ethical issues and considerations. Frameworks such as the European AI Act, the European Medical Devices Regulation, the European Health Data Space can and should be ultimately implemented with this objective in mind, thus increasing overall trust in digital means.

Invest in cost-effective mental health innovation with positive return on investments for health systems and increasing access to care—digital tools can provide solutions to provide timely care in a cost-efficient manner also to otherwise difficult to reach population (e.g., in “medical desert” areas or to citizens with more limited access to traditional care). Digital innovation should be supported in thriving environment offering on adequate and flexible support for innovation, while ensuring careful evaluation and analysis of the benefits to facilitate advancement of the most effective and secure tools.

## 3 Discussion

This paper proposes a holistic, personalised AI-based mHealth approach to preventive mental healthcare in youth, placing user needs, cost-effectiveness, and implementation at the forefront. While technological advancements in AI and digital health are progressing rapidly, their integration into routine care—especially for adolescents—remains limited. Major barriers include insufficient long-term evidence, unequal access, and fragmented implementation environments.

AI-powered mHealth tools offer great promise in empowering adolescents through personalised feedback, increased health literacy, and early recognition of mental health issues. To ensure uptake and impact, such tools must be co-developed with youth, tailored to diverse socio-economic contexts, and supported by policies that address literacy, accessibility, and safe innovation.

Scalability and relevance depend on user-centred design and integration into broader healthcare ecosystems. Preventive interventions can significantly reduce societal costs and health disparities, particularly among high-risk youth. However, success requires cross-sector collaboration, ethical oversight, and ongoing evaluation to realise sustainable, equitable impact in global mental health systems.

Overall, age-appropriate tools that empower young individuals and families to proactively manage their physical and mental health could foster lifelong healthy habits from an early age, leading to healthier adult populations and reduced healthcare costs. However, several challenges need to be considered.

### 3.1 Challenges

The adoption, development, and implementation of a holistic AI empowered personalised approach in the field of physical and mental prevention and intervention in adolescents and young people will contribute widely to reduce the burden of health problems and improve citizen’s quality of life and well-being. However, it also entails important challenges. In the following, we describe the most important limitations.

#### 3.1.1 High drop-out rates during intervention

Drop-out rates are high in digital self-help interventions [up to 85%; (101, 113)]. Personal engagement and relevance can be increased by the named participatory approach and individualised feedback, which has been shown to substantially decrease drop-outs in earlier projects (95, 102). Further, trials need to best be monitored externally, ensuring high user engagement and data quality.

#### 3.1.2 Limited public acceptance and uptake by the target population

The target group as end-users are central to all stages of the development of an intervention to enhance acceptance and to reduce negative attitudes. An AI-based mHealth tool can be translated into various languages and undergo cultural and regional adaptation. With an open-source approach, low-cost usage is possible if nations cover hosting and support expenses. User acceptance and low uptake rates of digital interventions represent common problems leading to high drop-out rates. Using participatory approaches by including the target group in all stages of the development and the evaluation process shows a promising way to reach more positive attitudes towards the mHealth solutions, and to enhance user acceptance (43). Another important issue involves cultural and regional adaptations, including translation to different languages and addressing individuals with different cultural backgrounds (114). Attention must be paid to ensure an open-source approach allowing adaptations in an easy way. However, participatory research, user-centred design, and cultural adaptation require a high amount of financial and temporal resources that should already be considered when planning digital projects. Nevertheless, the involvement of these features represent indispensable components for the development and implementation of digital interventions and are recommended in guidelines and frameworks for the development of e-health interventions (43). Involving institutions in different countries to cover and support expenses is recommendable at all stages of the development.

#### 3.1.3 Ethical and legal issues related with social disparities

Prevalence rates of youth psychopathology have increased in recent years, with underserved youth (e.g., racial/ethnic minorities, rural, and sexual minorities) disproportionately affected due to various risk factors. Individuals in these communities face significant barriers to accessing mental health services, such as stigma and high costs, which hinder their ability to support their mental health needs. (115). Digital mental health interventions could help overcome these barriers by providing accessible, culturally sensitive, and evidence-based resources to equip adolescents with the tools needed to support their well-being. To address potential disadvantages for lower socioeconomic status (SES) groups and ensure proper consent from underage populations, solutions must be developed with a focus on affordability, enabling access regardless of socio-economic background. For digital health tools to be safely and equitably integrated across all groups, alignment with the Digital Supply Act proposed by the European Parliament is essential. This includes ensuring CE marking, certification as medical devices, and system interoperability. By building trust and reducing digital literacy disparities, the goal is to enhance adherence to effective health interventions and ultimately improve health outcomes.

#### 3.1.4 Potential side effects of ecological momentary assessments

Measurement reactivity, the phenomenon where individuals’ behaviours or responses change due to heightened awareness from ongoing data collection, can manifest in various ways. For instance, in psychiatric populations, frequent engagement with symptoms through self-reports may impact symptom occurrence, as seen in disorders like binge eating, addiction, and trauma-related memories (e.g., repeatedly prompting smokers about their urge to smoke may increase the frequency of smoking cigarettes) (116–119). These effects, influenced by assessment frequency, regularity, and duration (120), along with patient characteristics like symptom severity (121), raise questions about potential adverse effects of monitoring applications, such as symptom deterioration. Qualitative studies highlight concerns about excessive focus on illness, potentially compromising autonomy (117). Therefore, further research and multimodal approaches are needed to understand and address these potential side effects.

#### 3.1.5 Potential risks in AI usage

AI is known to come with several risks, inlcuding limited explainability, potential bias and hence resulting limited fairness across user groups and interests, potential privacy breaches, potential miss-classification, potential attacks, e.g., by other AI targeting data safety or outcomes. It may also raise ethical questions such as whether energy invested into training and partially also inference of according models is justified by the usage, labelling of (training) data was carried out under ethically appropriate conditions, and further more (122). Another key trade-off is the need to collect large amounts of personal data from underage populations for AI models. As a result, ensuring robust data security, transparency, and responsible data management is crucial to maintaining safety and fostering trust in digital health interventions.

### 3.2 Summary and recommendation

In summary, the field of AI in youth mental healthcare is advancing rapidly, yet these innovations are not being sufficiently applied to reduce the growing gap in prevention and early intervention. There is widespread agreement that mHealth solutions require higher standards—including improved adherence, robust cost—benefit evaluations, and integration into routine care. To move forward effectively, we propose the following core recommendations for the development, evaluation, and implementation of AI-based mHealth interventions for youth mental health:

**Adopt holistic, transdiagnostic, and personalised approaches** that reflect the complexity of youth mental health and its interaction with physical, emotional, social, and environmental factors. Design interventions using **participatory, youth-centred co-creation processes**, involving adolescents, families, and mental health professionals from the earliest stages of development. **Ensure transparency, safety, and fairness of AI algorithms** through use of diverse training datasets, explainable models, and continuous validation in real-world settings. Provide **real-time, personalised feedback using smart active and passive data** (e.g., physical activity, mood patterns) to support behaviour change, increase self-awareness, and enhance health literacy. **Evaluate long-term impact with a focus on sustained behaviour change, cost-effectiveness, and equity** across different socio-economic and cultural contexts. **Collaborate with policymakers** to build enabling environments—through ethical frameworks, digital literacy efforts, and sustainable funding and reimbursement models. **Design for scalability and equity**, especially to reach underserved populations who are often excluded from conventional prevention efforts, thereby addressing the “prevention dilemma.”

Through these principles, AI-based mHealth tools can empower adolescents to monitor and improve their own mental and physical well-being, while also lowering barriers to early care access. Such tools can function as both stand-alone self-help interventions and as supportive complements to professional healthcare. In doing so, they offer the potential not only to reduce the individual and societal burden of mental illness, but also to promote long-term resilience and health equity among future generations.

## 4 Conclusion

We proposed an AI empowered personalised approach that could have the form of a holistic, individualised, AI-based mHealth preventive intervention for mental disorders and health behaviour featuring early and precise mental health status assessment for adolescents. Such an intervention would ensure continuous monitoring by smart active and passive assessment of digital biomarkers, covering physical and mental health. We outlined the necessity to evaluate the cost-effectiveness through multi-site, prospective randomised controlled trials across multiple countries, valuating the perspective of people with lived experience in a participatory approach on all levels of the scientific process. Implementation science should focus on the identification of barriers and facilitators of scaled application, and devise strategies for dissemination and sustainable health support across Europe.

By using this AI-driven prevention approach, we aim to help people improve their health and well-being in real, noticeable ways. Inequalities in healthcare may also be reduced by e.g., providing accessible and cost-efficient health services. Supporting healthier lifestyles may empower individuals to make better choices, live longer and healthier lives, lower healthcare costs, and stay active as they age—contributing to a more holistic view of well-being.

## Data Availability

The original contributions presented in the study are included in the article/Supplementary Material, further inquiries can be directed to the corresponding author/s.
